# Depressive Symptoms in Dementia Caregivers in Puerto Rico: The Role of Caregiver Burden and Symptoms of Dementia

**DOI:** 10.1093/geroni/igaf122.1502

**Published:** 2025-12-31

**Authors:** Junyub Lim, Ross Andel, Frank Puga, María Aranda, Maricruz Rivera-Hernandez, Ana Luisa Davila-Roman, Michael Crowe

**Affiliations:** Arizona State University, Phoenix, Arizona, United States; Arizona State University, Arizona State University, Arizona, United States; University of Alabama at Birmingham, Birmingham, Alabama, United States; University of Southern California, Los Angeles, California, United States; Brown University School of Public Health, Providence, Rhode Island, United States; University of Puerto Rico, San Juan, Puerto Rico, United States; University of Alabama at Birmingham, Birmingham, Alabama, United States

## Abstract

**Background/Objectives:**

Dementia is more prevalent in Puerto Rico than on the U.S. mainland, increasing demands for caregiving. However, research on the mental health impact of dementia caregiving among island Puerto Ricans is sparse. We examined caregiver burden and depressive symptoms among Puerto Rican dementia caregivers and a potential moderating role of behavioral and psychological symptoms of dementia (BPSD).

**Methods:**

Using data from the Puerto Rican Elder: Health Conditions (PREHCO) study (Wave 3), 132 caregivers of PREHCO participants completed an ancillary data collection, of whom 101 were dementia caregivers with complete data. Measures included the 15-item Geriatric Depression Scale (depressive symptoms), Neuropsychiatric Inventory Questionnaire (NPI-Q; BPSD), and Zarit Burden Interview (ZBI-6; caregiver burden).

**Results:**

Average caregiver age was 63±10 years and 77% were women. Exploratory factor analysis yielded two BPSD factors— Emotional Dysfunction/Behavioral Instability and Perceptual Disturbances/Apathy. In separate multivariable linear regression models adjusted for age, sex, and education, caregiver burden (standardized coefficient [β]=0.53, p<.001) was positively associated with depressive symptoms, as was Emotional Dysfunction/Behavioral Instability (β = 0.54, p<.001) and Perceptual Disturbances/Apathy (β = 0.55, p<.001). Emotional Dysfunction/Behavioral Instability (β = 0.20, p=.037) and Perceptual Disturbances/Apathy (β = 0.26, p=.009) moderated this association such that the caregiver burden-depressive symptoms link grew significantly stronger as BPSD increased.

**Discussion:**

These findings indicate that BPSD may influence caregiver mental health in Puerto Rican dementia caregivers, and that the effect of BPSD on this association follows a non-linear pattern. This highlights the need for tailored interventions to alleviate caregiver burden and depression based on managing BPSD in the care recipient with dementia.

